# Field Evaluation of Mobile Molecular Differential Tests in DRC and Nigeria

**DOI:** 10.1093/ofid/ofaf630

**Published:** 2025-10-08

**Authors:** Martin Faye, Sheila Makiala-Mandana, Moussa Moïse Diagne, Oumar Faye, Susanne Boehlken-Fascher, Jonas Kissenkoetter, Jean-Jaques Muyembe-Tamfum, Steve Ahuka-Mundeke, Placide Mbala-Kingebeni, Patient Okitale-Talunda, Gracia Kashitu Mujinga, Marc-Antoine de La Vega, Gary Kobinger, Oyinloye Oladapo Elijah, Adeleye Solomon Bakarey, Raifu Muideen Kolawole, Olusegun George Ademowo, Cheikh Tidiane Diagne, Amadou Alpha Sall, Ahmed Abd El Wahed, Ousmane Faye, Manfred Weidmann

**Affiliations:** Virology Department, Institut Pasteur de Dakar, Dakar, Senegal; National Institute for Biomedical Research, Kinshasa, Democratic Republic of Congo; Medical Biology Department, Faculty of Medicine, University of Kinshasa, Kinshasa, Democratic Republic of the Congo; Virology Department, Institut Pasteur de Dakar, Dakar, Senegal; Virology Department, Institut Pasteur de Dakar, Dakar, Senegal; Microbiology and Animal Hygiene, University of Goettingen, Goettingen, Germany; Microbiology and Animal Hygiene, University of Goettingen, Goettingen, Germany; National Institute for Biomedical Research, Kinshasa, Democratic Republic of Congo; Medical Biology Department, Faculty of Medicine, University of Kinshasa, Kinshasa, Democratic Republic of the Congo; National Institute for Biomedical Research, Kinshasa, Democratic Republic of Congo; Medical Biology Department, Faculty of Medicine, University of Kinshasa, Kinshasa, Democratic Republic of the Congo; National Institute for Biomedical Research, Kinshasa, Democratic Republic of Congo; Medical Biology Department, Faculty of Medicine, University of Kinshasa, Kinshasa, Democratic Republic of the Congo; Medical Biology Department, Faculty of Medicine, University of Kinshasa, Kinshasa, Democratic Republic of the Congo; Medical Biology Department, Faculty of Medicine, University of Kinshasa, Kinshasa, Democratic Republic of the Congo; Department of Microbiology and Immunology, University of Texas Medical Branch, Galveston, Texas, USA; Global Urgent Advance Research and Development, Batiscan, Quebec, Canada; Department of Microbiology and Immunology, University of Texas Medical Branch, Galveston, Texas, USA; Global Urgent Advance Research and Development, Batiscan, Quebec, Canada; Institute for Advanced Medical Research and Training, College of Medicine, University of Ibadan, Ibadan, Nigeria; Institute for Advanced Medical Research and Training, College of Medicine, University of Ibadan, Ibadan, Nigeria; Institute for Advanced Medical Research and Training, College of Medicine, University of Ibadan, Ibadan, Nigeria; Institute for Advanced Medical Research and Training, College of Medicine, University of Ibadan, Ibadan, Nigeria; Virology Department, Institut Pasteur de Dakar, Dakar, Senegal; DIATROPIX, Institut Pasteur de Dakar, Dakar, Senegal; Virology Department, Institut Pasteur de Dakar, Dakar, Senegal; Microbiology and Animal Hygiene, University of Goettingen, Goettingen, Germany; Institute of Animal Hygiene and Veterinary Public Health, Leipzig University, Germany; Virology Department, Institut Pasteur de Dakar, Dakar, Senegal; Institute of Aquaculture, University of Stirling, Stirling, Scotland, UK; Medizinische Hochschule Brandenburg Theodor Fontane, Senftenberg, Germany

**Keywords:** arboviruses, differential testing, fever of unknown origin, mobile suitcase laboratory, neglected tropical diseases, recombinase polymerase amplification

## Abstract

**Background:**

Accurate and timely differential diagnoses are a challenge for health care, particularly in infrastructure-poor settings.

**Methods:**

To investigate fevers of unknown origin in Africa, a mobile suitcase laboratory was deployed to DRC and Southwest Nigeria to support the control of the 2018–2020 Ebola virus disease outbreak in North-Kivu and Ituri provinces (DRC) and to provide a point-of-need solution for malaria confirmation during the dry season, respectively.

**Results:**

In DRC, the samples were tested for Ebola virus and the differentials *Plasmodium falciparum*, *Salmonella enterica*, yellow fever virus, Dengue virus, and chikungunya virus. In Southwest Nigeria, the samples were not tested for Ebola virus but were tested for the same differentials and additionally for *Rickettsia* spp., *Leptospira,* and *Streptococcus pneumoniae. Plasmodium falciparum* was detected in 23% (n = 192) and 47% (n = 88) of cases, respectively, and *Salmonella enterica* was detected in only 1 case in each cohort.

**Conclusions:**

The etiological agents circulating in febrile patients in Sub-Saharan Africa and the true incidence of neglected tropical diseases are still underestimated.

## Teaser

Field testing of a differential molecular test panel in DRC and Nigeria indicates possibly circulating aetiological agents in febrile patients in Sub-Saharan Africa are still underestimated.

## Graphical Abstract

**Graphical Abstract:**
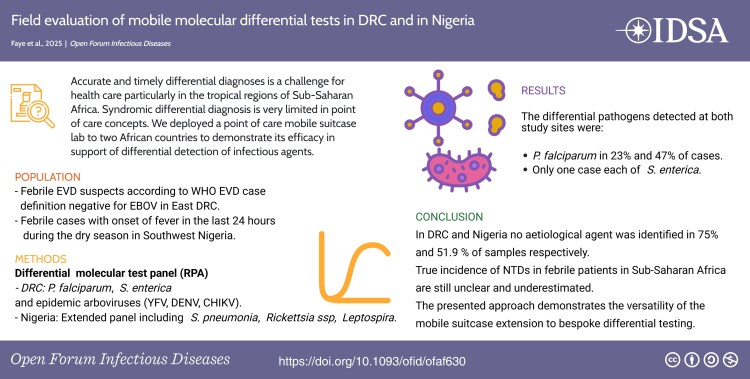
This graphical abstract is also available at Tidbit: https://tidbitapp.io/tidbits/infective-endocarditis-and-antimicrobial-timing-a-case-for-delay?utm_campaign&equals;tidbitlinkshare&utm_source&equals;IO

The 2018–2020 Ebola virus disease (EVD) outbreak in the east of the Democratic Republic of Congo (DRC) was associated with >3200 confirmed cases, including 56% female and 28% children under 18 years old [1]. Confirmation of positive cases relied on testing using a specific reverse transcription polymerase chain reaction (RT-PCR) assays targeting the viral nucleoprotein (NP) and glycoprotein (GP), Xpert Ebola Assay (GeneXpert Instrument Systems; Cepheid, Sunnyvale, CA, USA).

Malaria remains the primary cause of acute undifferentiated fever (or fever of unknown origin) in children across West Africa [2]. As the burden of this illness declines on the continent [3, 4] and parasitological testing increases [5], a growing number of patients who would have been treated for malaria are now being diagnosed with different conditions [6]. Managing these patients appropriately still presents challenges for health care providers [7].

Despite the satisfactory performance and availability of malaria rapid diagnostic kits (mRDTs), there is evidence of antimalarial treatment of patients with negative mRDTs undermining the benefits of diagnostic screening [8]. In some contexts, the decrease in antimalarial consumption after introducing mRDTs was accompanied by increased use of antibiotics [8, 9], raising concerns about potential emergence of antibiotic resistance.

A mobile suitcase laboratory for Ebola virus (EBOV) point-of-care detection at Ebola treatment centers was successfully implemented in Guinea during the large EVD outbreak in West Africa in 2014–2015. It was shown that isothermal amplification (recombinase polymerase amplification [RPA]) could be efficiently used to test suspected EVD cases, and local teams were trained in and successfully deployed this rapid method [10]. In the EVD outbreak in Guinea, up to 90% of patients fitting the case definition for EVD tested negative for EBOV, but these individuals were rarely diagnosed for differentials. Malaria and other diseases went underdiagnosed, which most likely led to more deaths from these infectious agents. Another important aspect is that the fear of patients of having Ebola while they are suffering from other diseases must be overcome by providing correct and accurate diagnosis of the type of pathogens involved in good time.

Accurate and timely differential diagnosis is a challenge for health care, particularly in the tropical regions of Sub-Saharan Africa. Syndromic differential diagnosis is very limited in point-of-care concepts. We deployed a point-of-care mobile suitcase lab to 2 African countries to demonstrate its efficacy in support of differential detection of infectious agents.

To investigate fevers of unknown origin in Africa, our mobile suitcase laboratory was deployed to East DRC and Southwest Nigeria to support control of the 2018–2020 EVD outbreak in North-Kivu and Ituri provinces (DRC) and to provide a point-of-need solution for malaria confirmation during the dry season, respectively.

## METHODS

### Study Design

A prospective cross-sectional study was designed to identify the etiology of febrile cases fitting the World Health Organization (WHO) EVD case definition [11] who were negative for EBOV in East DRC. Differentials tested were the main “suspected epidemic febrile” differentials, *P. falciparum* and *Salmonella enterica,* as well as epidemic arboviruses (*Orthoflavivirus flavi* [yellow fever virus {YFV}], *Orthoflavivirus denguei* [Dengue virus 1–4 {DENV}], and *Alphavirus chikungunya* [Chikungunya virus {CHIKV}]).

In Southwest Nigeria, the main study site was a primary health care center in Abanla, located in the outskirts of Ibadan, Southwest Nigeria. The study was done in the dry season, which is known for a relatively low prevalence of malaria. Patients presenting with onset of fever within the last 24 hours were recruited into the study upon consent. By default for every sample, a blood smear was stained with Giemsa and screened by conventional microscopy for detection of *Plasmodium* using the WHO protocol [12]. The differential RPA panel described above was extended to include *Streptococcus pneumonia* as Nigeria in particular suffers from the second highest burden globally [13, 14]. Also included were *Rickettsia* spp. and *Leptospira* as high incidences have been reported from tropical regions including Nigeria for both pathogens [15, 16].

Calibration and validation of all assays were performed at the University of Leipzig and the Institut Pasteur de Dakar before deployment, in comparison with quantitative polymerase chain reaction (qPCR), to ensure accurate and reliable results. In the field, indeterminate results were addressed by retesting samples.

### Ethical Approval

In collaboration with staff from the Ebola Treatment Unit (ETU) in the Katwa district (North-Kivu province, East DRC) all EVD-negative cases were included (national ethical approval assigned number: ESP/CE/098/2019). Teams at the mobile suitcase laboratory worked closely with the clinical staff of the health care services facility in Southwest Nigeria, where all febrile patients were invited to participate in the study (national ethical approval UI/UCH ethics committee assigned number: UI/EC/17/0005). Use of a patient consent form procedure was a condition for both approvals.

### RPA Assays

A recombinase polymerase assay (RPA) for the detection of *Plasmodium falciparum* was adapted for fluorescent RPA detection (Table 1) [17]. The RPA kit TwistGlow *Salmonella* kit (Twist Dx, Maidenhead, UK) was used for detection of *Salmonella enterica*. The remaining RPAs were all previously described: *Streptococcus pneumonia* [18], *Rickettsia* spp. [19], *Leptospira* [20], YFV [21], DENV [22], and CHIKV [23]. Additionally, an RPA for detection of the human β-actin gene was developed and used as a control to test for sampling and extraction quality and as a generic RPA-positive control (GPC) (Table 1). No template controls (NTCs) and strict procedures such as dedicated lab coats, proper use of personal protective equipment, and meticulous decontamination of the bench and the device were implemented to avoid contamination.

**Table 1.** Oligonucleotides

Organism	Target Gene	Oligonucleotide Designation	Oligonucleotide Sequence 5′–> 3′	Analytical Sensitivity [Molecules/Reaction] (95% Probit)
*P. falciparum*	18S SSU	PF RPA P	gTgTTTgAATACTACAgCATggAATAACAAA**-BTF-**gAATAAgCTAATTATT-P	1.88
Generic positive	Human Beta-actin	ACTB RPA FP	gTCCACCTTCCAgCAgATgTggATCAgCAA	116
Control (GPC)	…	ACTB RPA RP	TgTCAAgAAAgggTgTAACgCAACTAAgTC	
	…	ACTB-RPA P	gCAggAgTATgACgAgTCCggCCCCTCCA**-BTF**-CCACCgCAAATgCTT-P	

Probit 95% values were obtained by testing quantitative in vitro dsDNA standards (n = 6) on the Axxin device.

Abbreviations: BTF, B—thymidine nucleotide carrying Blackhole quencher 1, T—tetrahydrofuran spacer, F—thymidine nucleotide carrying Fluorescein; GPC, generic recombinase polymerase amplification–positive control; Phosphate, 3′phosbate to block elongation.

All of the assays were implemented in the existing mobile suitcase laboratory concept [24] to allow for testing of the major confounding epidemic differentials in an EVD outbreak and a malaria-endemic context. The amplification concept is sequential, with all extracted samples first being tested for the human ß-actin gene followed by parallel RPA amplification using the 8-strip RAA kits from the *Jiangsu Qitian* Gene Biotechnology company (China), with each tube targeting 1 of the individual differentials [25].

### Nucleic Acid Extraction

In DRC, RNA was extracted from blood samples using the QIAamp Viral RNA mini kit (QIAGEN, Hilden, Germany) according to the manfuacturer`s instructions. In DRC and Nigeria, total nucleic acid extraction was done from serum samples using the SpeedExtract kit (Qiagen, Hilden, Germany) according to the manufacturer`s instructions. Briefly, 200 µL of SL-Buffer, 30 µL of bead suspension, and a 20-µL serum sample were mixed in a 2-mL tube and vortexed. The mix was incubated at 95°C in a small heat block and repetitively vortexed and reinserted every 2 minutes during a 10-minute incubation period. Thereafter, the tube was briefly spun down and placed into a magnetic stand for 1 minute. The supernatant was pipetted into a new tube, and 5 µL of supernatant was used in the RPA reaction. Five microliters of eluate each was tested in the respective differential RPA assays. The RPAs were performed in an 8-strip format with dried primer and probe mixes for each reaction as previously described [26].

### Data Presentation and Statistical Analysis

Participants were categorized according to age, following the same pattern of the population's statistics and demography guidelines (<15, 16–30, 31–45, 46–60, and >60 years). Positive results were classified according to the category of the pathogen (protozoa, bacteria, virus). Most analyses consist of simple proportions of positive samples (ie, samples that indicated the presence of a pathogen by total samples analyzed).

## RESULTS

### Differential Testing Results in DRC

In DRC, a total of 220 samples were tested by the differential RPA panel from patients with a median age of 20 years (age range, 0–98 years) and a sex ratio of 1.02. Reference testing by GeneXpert targeting 2 EBOV genes scored 119/220 EBOZV-negative and 1/220 EBOZV-positive. With the differential RPA panel, 192 (87%) were positive by GPC; failure of a GPC signal in 28 samples indicated bad sampling efficiency and low quality of human sample material. Forty-six of 192 samples scored positive for *P. falciparum* (23%), and 1 was positive for *Salmonella* sp. (0.5%). One hundred forty-five samples were negative for all parameters (75%). In addition, the EBOZV-positive samples tested negative for all parameters.

To confirm the RPA results, the 145 remaining samples were reference-tested by qRT-PCR for *P. falciparum* (LightMix Modular Plasmodiium genus [530], Roche, Germany), CHIKV [27], and DENV [28] at Institut National de Recherche Biomédicale (INRB), to which the samples had been transferred. All tested negative for DENV and CHIKV, thus confirming the negative RPA results. Only 5/110 tested positive for *P. falciparum* by PCR (cycle threshold [Ct] range, 19–8 −29.3). Three of these were positive by RPA, and 3 scored negative by RPA (Ct 25.3 [2×], > Ct 35.4 [3×]).

Surprisingly, all other infectious agents tested negative by PCR, leaving 41 samples *P. falciparum* positive by RPA but negative by PCR.

All RPA positives were also positive in the IPC RPA, indicating that the RPA reaction worked correctly. It is quite possible that there is a major sensitivity issue with the malaria qPCR used, which appears not to be sensitive enough for *P. falciparum*; alternatively, the oligonucleotides used in qRT-PCR need to be cross-checked against indigenous *P. falciparum* target gene sequences.

### Differential Testing Results in Nigeria

In Southwest Nigeria, 88 blood samples were tested from patients (age range, 1–63 years), of whom 41 (47%) tested positive using the *P. falciparum* RPA assay. Only 1 patient sample (1.1%) was positive using the *Salmonella* RPA assay. None of the other pathogens were detected. The positivity rate for *P. falciparum* found in Nigeria was significantly higher than that obtained in DRC (*P* < .0001). All RPA-positive cases were confirmed by smear test microscopy.

## DISCUSSION

A mobile suitcase laboratory for point-of-care differential testing of probable etiologies of non-EVD illness was successfully implemented near the ETU in the Katwa district (North-Kivu province) during the 2018–2020 EVD outbreak in DRC and in a malaria-endemic context in Nigeria for rapid confirmation of etiologies in febrile patients. The climate of the Kivu region in DRC is described as humid-tropical tempered by altitude. A growing population including a large displaced population due to the ongoing civil war is seeing a constantly rising malaria incidence, which was calculated at 15 501/100 000 in 2015 [29]. Southwest Nigeria has a tropical climate with significant rainfall and a short dry season. Malaria is endemic, with stable ongoing transmission and a prevalence of >94% recently recorded in a study of 300 patients visiting hospitals [30]. Given these conditions, the main differential pathogen detected at both study sites was *P. falciparum* in 23% and 47% of febrile EVD-negative cases and of febrile suspected malaria cases, respectively. Not surprisingly, a roughly 2-fold higher positivity rate was detected in the cohort of suspected malaria cases as opposed to the cohort of EBOV-negative cases in DRC.

Isothermal amplification RPA could be efficiently used to perform differential testing of EVD-negative cases and suspected malaria cases. *P. falciparum* was detected in 23% and 47% of cases, respectively. This demonstrates once more that molecular detection and in particular isothermal amplification methods can be a useful tool for malaria diagnostics [31]. A recent meta-analysis of 29 studies on the use of PCR and LAMP vs RDTs and microscopy for malaria diagnostics in Ethiopia demonstrated this conclusively [32].

The discrepancy between the commercial PCR kit results and the RPA results could not be followed up as we had no access to the oligonucleotide sequences of the commercial kit. However, once again it was made clear that for all molecular assays target erosion needs to be constantly monitored. This is now a postmarket surveillance requirement of the In vitro Diagnostic Regulation (IVDR) regulations in Europe. A better PCR reference test needs to be identified in order to determine potential RPA false-positive rates.

In both cases, only 1 *Salmonella*-positive case was identified (0.5% and 1.1%). This may be due to the known low pathogen concentration in blood in acute cases [33]. Another study that tested 741 febrile children in Guniea-Bissau with a PCR panel and scored 27/544 positive hits for *Salmonella enterica* (4%) confirms the low detection rate in our study. The slightly elevated value may be due to the target population being exclusively children <5 years of age and the larger sample size [6].

Arbovirus epidemics can have a significant impact, and in 2023 Burkina Faso experienced a Dengue outbreak with 154 867 suspected cases, 70 433 confirmed by rapid diagnostic tests, and 709 recorded deaths [34].

In DRC and Nigeria, no etiological agent was identified in 75% and 51.9% of samples, respectively. The study in febrile children in Guinea-Bissau yielded 27% negatives, indicating that, as commonly circulating arboviruses were ruled out in all 3 studies, current evidence about possibly circulating etiological agents in febrile patients in Sub-Saharan Africa is still underestimating the true incidence of neglected tropical diseases (NTDs). Although Sub-Saharan Africa recorded the most significant decline in NTD cases over the past 3 decades [35], NTDs continue to exert an immense toll, rivalling major infectious and noncommunicable diseases, particularly in Western Saharan Africa, which has the highest incidence of NTDs globally [35]. Thus, sustained investments in strengthening local health systems [36] will be crucial to achieving the goal of 90% reduction in impact and transmission of NTDs by 2030 [37].

In addition, local teams were trained in and successfully deployed this rapid method (Supplementary Figure 1).

Although malaria rapid diagnostic tests play a crucial role in increasing access to diagnosis in regions where high-quality microscopy is not feasible [38], their effectiveness is directly influenced by the density of parasites. Recent research showed a sensitivity >95% when the density is ≥400 parasites/μL and nearing 100% at a density of 4000 parasites/μL, with negative predictive values of 88% during the rainy season and 95% in the dry season [39]. This project trained teams in the DRC and Nigeria and expanded the RPA testing capacity to the differentials for EVD and malaria recommended by the WHO [1, 12]. Through this successful cooperation, the DRC and Nigeria teams are now able to provide field diagnostic response capacity for infectious disease outbreaks. The collaboration with teams from West Africa and Central Africa that are already proficient in the use of the laboratory suitcase has helped to build a stronger network of collaborating African outbreak response teams [40].

The presented approach demonstrates the versatility of the mobile suitcase extension to bespoke differential testing.

## Supplementary Material

ofaf630_Supplementary_Data

## Supplementary Data

Supplementary materials are available at *Open Forum Infectious Diseases* online. Consisting of data provided by the authors to benefit the reader, the posted materials are not copyedited and are the sole responsibility of the authors, so questions or comments should be addressed to the corresponding author.
