# Correction to: Field Evaluation of Mobile Molecular Differential Tests in DRC and Nigeria

**DOI:** 10.1093/ofid/ofaf738

**Published:** 2025-12-12

This is a correction to: Faye M, Makiala-Mandana S, Diagne MM et al. Field Evaluation of Mobile Molecular Differential Tests in DRC and Nigeria, *Open Forum Infectious Diseases*, Volume 12, Issue 10, October 2025, ofaf630, https://doi.org/10.1093/ofid/ofaf630.

In the original publication of this article, *Leptospira* was erroneously referred to as *Leptospira donovani* in the RPA Assays subsection of Methods. This has been corrected in the version of the article currently available online to prevent confusion with *Leishmania donovani*, which was not reviewed in this article’s evaluation of mobile molecular differential tests.

