# Supplementary material for: Field Evaluation of Mobile Molecular Differential Tests in DRC and Nigeria
Source: Open Forum Infect Dis. 2025 Oct 8;12(10):ofaf630. doi: 10.1093/ofid/ofaf630 (PMC12569597; doi:10.1093/ofid/ofaf630)
Supplement: ofaf630_Supplementary_Data [file ofaf630_supplementary_data.docx]

**Supplementary materials**


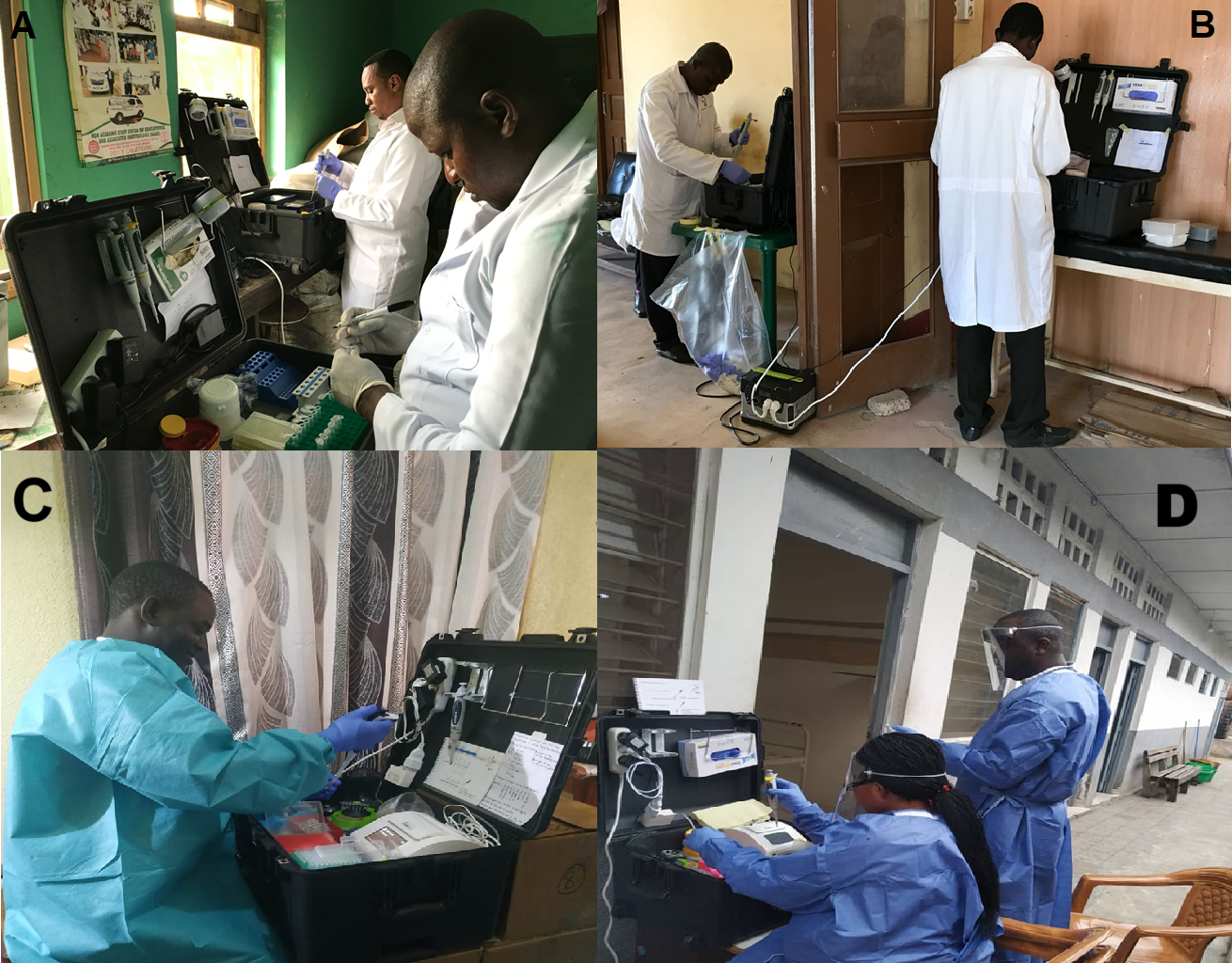


Supplementary Figure 1: Deployment of the mobile suitcase laboratory for screening blood samples from patients with fever at (A) Idiayunre and (B) Abanla primary Health care centres, South-West Nigeria, and at (C and D) Katwa healthcare centre (North-kivu) in DR Congo.
